# A Multi-Scale Self-Supervision Approach for Bearing Anomaly Detection Using Sensor Data Under Multiple Operating Conditions

**DOI:** 10.3390/s25041185

**Published:** 2025-02-15

**Authors:** Zhuoheng Dai, Lei Jiang, Feifan Li, Yingna Chen

**Affiliations:** College of Science and Technology, Ningbo University, Ningbo 315300, China; 2311170002@nbu.edu.cn (Z.D.); jianglei2@nbu.edu.cn (L.J.); 2211170007@nbu.edu.cn (F.L.)

**Keywords:** early anomaly detection, imbalanced industrial time series, multiple operating conditions, self-supervised learning

## Abstract

Early fault detection technologies play a decisive role in preventing equipment failures in industrial production. The primary challenges in early fault detection for industrial applications include the severe imbalance of time-series data, where normal operating data vastly outnumber anomalous data, and in some cases, anomalies may be virtually absent. Additionally, the frequent changes in operational modes during machinery operation further complicate the detection process, making it difficult to effectively identify faults across varying conditions. This study proposes a bearing early anomaly detection method based on contrastive learning and reconstruction approaches to address the aforementioned issues. The raw time-domain vibration data, which were collected from sensors mounted on the bearings of the machinery, are first preprocessed using the Ricker wavelet transform to effectively remove noise and extract useful signal components. These processed signals are then fed into a BYOL-based contrastive learning network to learn more discriminative global feature representations. In addition, we design the reconstruction loss to complement contrastive learning. By reconstructing the masked original data, the reconstruction loss forces the model to learn detailed information, thereby emphasizing the preservation and restoration of local details. Our model not only eliminates the reliance on negative samples found in mainstream unsupervised methods but also captures data features more comprehensively, achieving superior fault detection accuracy under different operating conditions compared to related methods. Experiments on the widely used CWRU multi-condition-bearing fault dataset demonstrate that our method achieves an average fault detection accuracy of 96.97%. Moreover, the experimental results show that on the full-cycle IMS dataset, our method detects early faults at least 2.3 h earlier than the other unsupervised methods. Furthermore, the validation results for the full-cycle XJTU-SY dataset further demonstrate its excellent generalization ability.

## 1. Introduction

Bearings are critical components in many industrial machines, and even minor damage can gradually escalate, leading to significant malfunctions. By leveraging advanced early anomaly detection techniques, it becomes possible to detect the initial signs of bearing wear, cracks, or other forms of damage. This timely detection not only allows for timely maintenance but also contributes to a substantial reduction in both equipment downtime and maintenance costs [1,2,3].

In the realm of anomaly detection, methodologies are broadly bifurcated into supervised and unsupervised paradigms, predicated on the exigency for annotated data. Supervised anomaly detection techniques are contingent upon meticulously labeled datasets to discern the quintessence of normal and anomalous states for prognostic endeavors. During the pedagogical phase, these techniques harness labeled data to architect models that adjudicate nascent samples based on the assimilated patterns. A cohort of relatively orthodox supervised algorithms, such as support vector machines (SVMs) [4] and decision trees [5], are ensconced within this category. Nonetheless, these classical methodologies grapple with impediments in feature extraction. The prodigious evolution of deep learning techniques in recent epochs has ameliorated this deficiency and has been ubiquitously employed in anomaly detection tasks. Convolutional neural networks (CNNs) [6] and long short-term memory (LSTM) networks [7] epitomize two salient exemplars. CNN and LSTM epitomize quintessential neural network architectures and manifest prodigious prowess in feature extraction for spatial and sequential data, respectively. Savants have proffered pertinent methodologies [8,9,10,11] and have executed experiments on a panoply of predictable and unpredictable time-series datasets. The empirical evidence has evinced their robustness in both ephemeral and protracted time-series analyses, underscoring the efficacy of these deep learning-centric approaches in anomaly detection.

However, in the crucible of practical industrial applications, the procurement of labeled data is often beleaguered by challenges due to its exorbitant cost and temporally intensive nature [12,13]. Consequently, the deployment of supervised methods is circumscribed to scenarios where data are pre-labeled [14]. In riposte to these challenges, unsupervised anomaly detection methodologies have burgeoned as a pivotal research trajectory. These methodologies dissect data structures to pinpoint anomalies, thereby attenuating the reliance on labeled data [15]. They evince remarkable advantages in the incipient detection of bearing anomalies, especially when labeled data are sparse or arduous to procure [16].

To this end, a compendium of unsupervised methods has been cultivated. Huang et al. [17,18] introduced the first-kind tensor singular value decomposition (1K-FTSVD) and an adaptive signal decomposition technique christened second-kind tensor singular spectrum decomposition (2K-FTSSD). These innovations surmount the inherent limitations of traditional tensor SVD predicated on the n-mode product, as well as the quandaries of non-unique optimization results and non-pseudo-diagonal core tensors. They eclipse traditional methods in the incipient detection of anomalies. Kang et al. [19] propounded a method denominated DIDAD, predicated on autoencoders, which distills features from high-frequency signals and refines anomaly detection. Other unsupervised anomaly detection methods, such as USSCNN, a CNN-based model proffered by Song et al. [20], UODA, a model predicated on autoencoders and RNNs proposed by Lu et al. [21], and FDDA, a method based on deep neural networks (DNN) and LSTM posited by Lu et al. [22], all evince superlative performance in feature extraction and excel in manipulating disparate types of data.

Despite the strides made by unsupervised methods in vanquishing the challenges concomitant with the need for specialized knowledge and prior experience with labeled data, their implementation in veritable industrial milieus remains formidable. Particularly in the context of bearing anomaly detection, industrial samples often suffer from a pronounced imbalance in time-series data. Normal data are copious, while anomalous data are scarce or even nonexistent. This imbalance exacerbates the conundrum of model generalization, as the paucity of anomalous data stymies the model’s capacity to efficaciously learn the features of anomalies. Moreover, the vibration signal data of bearings under disparate operating conditions can exhibit substantial variations [23]. These variations can precipitate shifts in data distribution, which in turn undermine the model’s generalization capabilities. A model that excels under certain conditions may falter under others, rendering it intricate and challenging to directly apply extant unsupervised anomaly detection methods in authentic industrial settings.

To tackle the critical issues of data imbalance and variations in operating conditions in real industrial environments, we specifically devise a novel early anomaly detection model named ADBR (**A**nomaly **D**etection method, based on the **B**YOL structure and **R**econstructed structures), and the main contributions of this study are as follows:

(1) To address the issue of scarce or nonexistent abnormal samples in real industrial data, this paper presents an ADBR unsupervised model. Based on the BYOL contrastive structure, this model effectively trains the anomaly detection model while completely eliminating the reliance on negative sample pairs, which is common in other unsupervised models.
(2) To address the frequent variations in operating conditions, the proposed ADBR model learns global features while retaining and reconstructing local details, enabling it to focus on both the overall structure and local nuances of the signals. By combining global- and local-scale learning strategies, the model comprehensively captures data features under different operating conditions, achieving superior accuracy in bearing anomaly detection compared to related methods across various conditions.
(3) Experiments on the widely used CWRU multi-condition bearing fault dataset demonstrate that our method achieves an average fault detection accuracy of 96.97%. Moreover, the experimental results show that on the full-cycle IMS dataset, our method detects early faults at least 2.3 h earlier than the other unsupervised methods. Furthermore, the validation results for the full-cycle XJTU-SY dataset further demonstrate its excellent generalization ability.

The remainder of this paper is organized as follows. Section 2 provides a detailed description of the proposed method, and Section 3 describes the details of the experimental data used. Section 4 describes the data preprocessing method. Section 5 presents the specific experiments and analyzes the results. Section 6 summarizes the results of this study.

## 2. Methodology

ADBR primarily consists of three key components. First, we utilize the Ricker wavelet to process the original one-dimensional time-series data, which helps remove noise while extracting useful signal components. Next, we employ a BYOL-based network structure to extract global features. Bootstrap Your Own Latent (BYOL) model [24] trains without needing negative sample pairs, making it suitable for real-world industrial data. It focuses on bringing positive sample pairs closer together, emphasizing the relationships between samples rather than their absolute values. This strategy not only enhances robustness against noise and interference, which are common challenges in industrial settings, but also breaks the dependency on negative samples that is characteristic of other contrastive models. Additionally, the Ricker wavelet is highly sensitive to sharp changes and transient signals. Anomalies in industrial time-series data typically manifest as brief spikes. Therefore, we design a reconstructed structure to extract local features. The reconstruction loss compels the model to focus more on the detailed information of the data by comparing the original and reconstructed data. By integrating global- and local-scale learning strategies, the model can comprehensively capture data features for early anomaly detection of bearings under multiple operating conditions, thereby enhancing the precision of anomaly detection across diverse operational conditions.

**Figure 1:**
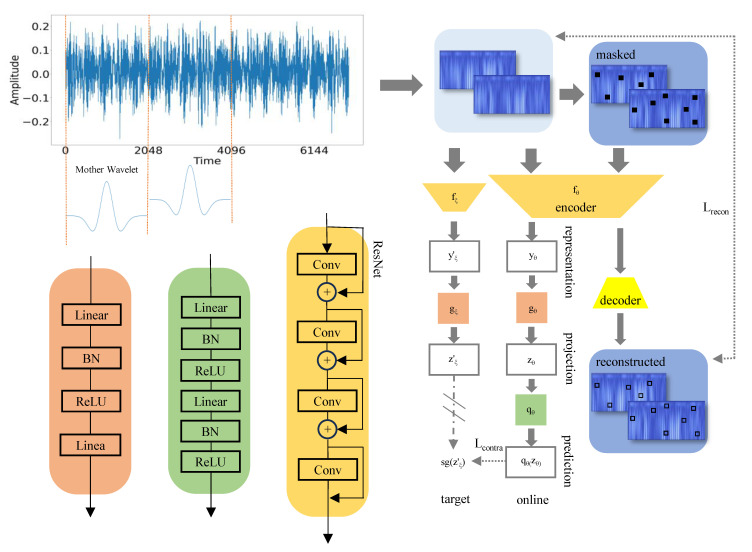
The detailed workflow: the three modules in the lower-left corner are expanded with the corresponding colors.

Figure 1 illustrates the detailed workflow, which consists of three parts: the Ricker wavelet transform, BYOL-based network structure, and reconstruction structure. Table 1 provides detailed notes on all the symbols in this section.

### 2.1. Ricker Wavelet Transform

For a given segment of the normal sample, the raw time-series data $D_{\mathrm{normal}}=\left[X_{1},X_{2},\ldots ,X_{n}\right]\in R^{t\times n}$, where each $X_{i}$ consists of

$$X_{i}={\left[x_{1i},x_{2i},\ldots ,x_{ti}\right]}^{T}\in R^{t},$$

The Ricker wavelet transform is defined as

$$\psi \left(t\right)=\left(1-\frac{t^{2}}{\sigma^{2}}\right)e^{-\frac{t^{2}}{2\sigma^{2}}}.$$

Applying the Ricker wavelet transform to each sample $X_{i}$ yields the corresponding time–frequency representation $P_{i}$:

$$P_{i}=X_{i}\times \psi \left(t\right).$$

Thus, we obtain set *P*, where $P=[P_{1},P_{2},\ldots ,P_{n}]$.

### 2.2. BYOL-Based Network Structure

After obtaining the time–frequency representation set *P*, we input it into $f_{\theta }$ to decode it into features *y* and $y^{\prime }$, which are subsequently fed into the online and target network structures, respectively. As shown in Figure 1, the network structure based on the BYOL model consists of online and target networks. The online network includes a projection head $g_{\theta }$ and prediction head $q_{\theta }$, whereas the target network contains a projection head $z_{\xi }^{\prime }$, where ξ is the exponential moving average of θ:

ξ←τξ+(1−τ)θ.

In the model implementation, we use ResNet-18 as the base model for the encoder, employing pretrained weights from PyTorch. The output dimension of the encoder is 512, whereas that of the online network projection head is modified to 2 for anomaly detection. The projection and prediction heads consist of a combination of linear and batch normalization layers. For each time–frequency representation $P_{i}$, the outputs of the online and target networks are denoted by $q_{\theta }\left(z_{\theta }\right)$ and $z_{\xi }^{\prime }$, respectively. Therefore, we have

$$L_{\mathrm{contra}}=\frac{1}{n}\sum_{i=1}^{n}{∥q_{\theta }\left(z_{\theta }\right)-z_{\xi }^{\prime }∥}_{2}^{2}.$$

### 2.3. Reconstruction Structure

To further enhance the performance of the model, we introduce the reconstruction loss. During training, $P_{i}$ is simultaneously masked to generate set $P_{i}^{\prime }$. The masked $P_{i}^{\prime }$ is input into the encoder and reconstructed back to the original time–frequency representation ${\hat{P}}_{i}$ by the decoder. The decoder is a simple, fully connected network that reconstructs the original image from the output of the encoder.

The reconstruction loss is calculated as follows:

$$L_{\mathrm{recon}}=\frac{1}{n}\sum_{i=1}^{n}{∥P_{i}-{\hat{P}}_{i}∥}_{2}^{2}.$$

### 2.4. Objective Function

The combination of contrastive learning and reconstruction loss utilizes two different training objectives, with the former focusing on the alignment of global features, and the latter emphasizing the recovery of local details. This dual-scale training strategy enhances the generalization and representation capabilities of the model, resulting in improved performance in various industrial scenarios. The overall objective function is given by

$$L=\alpha L_{\mathrm{contra}}+\left(1-\alpha \right)L_{\mathrm{recon}}.$$

where α is a hyperparameter used to balance the importance of the contrastive loss and reconstruction loss. The following Algorithm 1 demonstrates a more detailed process.
**Algorithm 1:** ADBR  **Input**: $D_{\mathrm{train}}\leftarrow 70\%\;\mathrm{of}\;D_{\mathrm{normal}}$  **Input**: $X_{i},X_{j}\leftarrow$ Randomly select two samples $X_{i}$ and $X_{j}$ from $D_{\mathrm{train}}$  **Output**: $g_{\theta }$  **Data**: Initialize: all parameters**_1._**   **Training:****_2._**   $P_{i}=X_{i}*\psi ,\;P_{j}=X_{j}*\psi$**_3._**   $z_{1}\leftarrow g_{\theta }\left(f_{\theta }\left(P_{i}\right)\right),\;z_{2}\leftarrow g_{\theta }\left(f_{\theta }\left(P_{j}\right)\right)$**_4._**   $z_{1}^{\prime }\leftarrow g_{\xi }\left(f_{\xi }\left(P_{j}\right)\right),\;z_{2}^{\prime }\leftarrow g_{\xi }\left(f_{\xi }\left(P_{i}\right)\right)$**_5._**   $L_{\mathrm{contra}}=\frac{1}{2}\left(∥q_{\theta }\left(z_{1}\right)-z_{1}^{\prime }{∥}_{2}^{2}+{∥q_{\theta }\left(z_{2}\right)-z_{2}^{\prime }∥}_{2}^{2}\right)$**_6._**   $L_{\mathrm{recon}}=\frac{1}{2}\left(∥P_{i}-{\hat{P}}_{i}{∥}_{2}^{2}+{∥P_{j}-{\hat{P}}_{j}∥}_{2}^{2}\right)$**_7._**   $L=\alpha L_{\mathrm{contra}}+\left(1-\alpha \right)L_{\mathrm{recon}}$

## 3. Experimental Setup

In the experimental section, we select three classic bearing datasets: the CWRU [25] abnormal bearing dataset, and the IMS [26,27,28] and XJTU-SY [29] full-cycle datasets. The datasets from CWRU, IMS, and XJTU-SY all contain rolling bearing data. The CWRU dataset contains the vibration signals of bearings under different operating conditions, making it suitable for testing the anomaly detection capability of the model across multiple conditions. The IMS and XJTU-SY datasets covered the complete degradation process of the bearings from normal to failure, allowing us to verify the performance of the model for early anomaly detection. To validate the accuracy of our method for early anomaly detection in bearings, we first use the classic CWRU abnormal bearing dataset to demonstrate our method’s ability to distinguish between positive and negative samples in various conditions, and then use the full-cycle bearing data to show the effectiveness and generalization ability of our method in detecting early anomalies. Table 2 presents the configuration of our training environment. Table 3 presents the training details of our model.

### 3.1. Preparation of Data

#### 3.1.1. Abnormal Bearing Dataset

**Figure 2:**
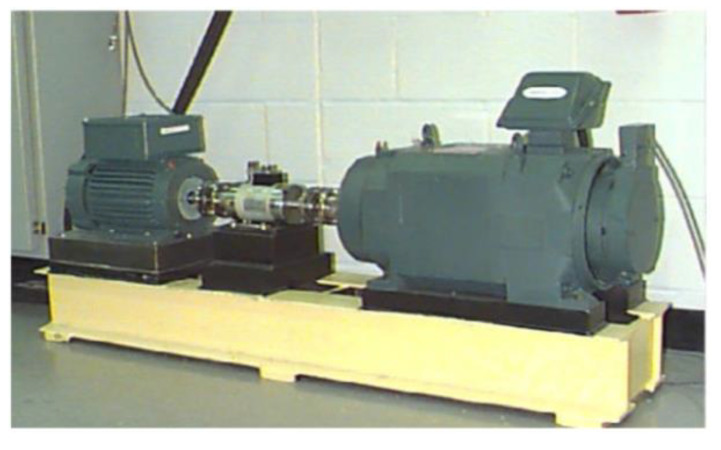
CWRU bench.

The CWRU dataset [30] contains vibration signals collected from motor bearings, covering various types of bearing faults. Its working platform is shown in Figure 2. The data are acquired under different operating conditions with corresponding loads of 0 horsepower, 1 horsepower, 2 horsepower, and 3 horsepower. The vibration signals are collected using accelerometers mounted on the bearing housing with a sampling frequency of 12 kHz. The operating conditions in the CWRU dataset are categorized into four types: (1) normal state, no faults; (2) inner race fault, where defects are present in the inner race of the bearing; (3) outer race fault, where defects are present in the outer race of the bearing; and (4) rolling element fault, where issues occur in the rolling elements of the bearing. In the fault states, the sizes of the bearing defects are categorized into three types: 0.007, 0.014, and 0.021, corresponding to different levels of fault severity. Table 4 lists all abnormal types.

#### 3.1.2. Full-Cycle IMS Bearing Dataset

**Figure 3:**
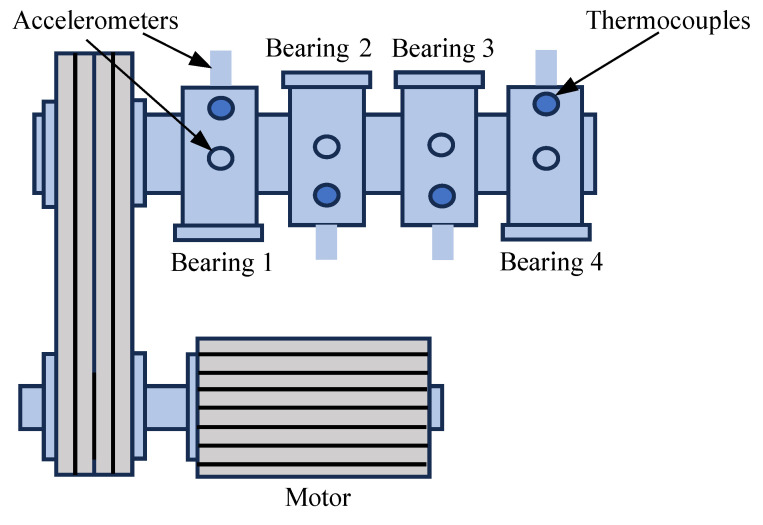
IMS bench.

The IMS dataset [26] contains vibration signal data spanning from normal operating conditions to the occurrence of bearing faults. Data are collected from four identical bearings driven by a motor. The motor speed is maintained at 2000 rpm, and a radial load of 6000 lbs is applied using a spring mechanism. The test platform is illustrated in Figure 3. In the first dataset, two high-sensitivity accelerometers are installed on each bearing housing, and 1 s of vibration signals is recorded every 10 min (the first 43 files are recorded every 5 min). This dataset includes 2156 files, with the first 1000 files representing normal operating conditions. At the end of the experiment, an inner race failure is detected in the third bearing, a rolling element failure occurs in the fourth bearing, and the remaining two bearings remain intact.

In the second dataset, only one high-sensitivity accelerometer is mounted on each bearing housing, and the recording interval is maintained at 1 s every 10 min. This dataset comprises 984 files, with the first 400 files representing normal conditions. By the end of the experiment, only the outer race of the first bearing fails, whereas the other three bearings remain healthy.

Both datasets have a sampling frequency of 20,480 Hz, with more detailed specifications provided in Table 5. This dataset offers extensive vibration signal information for evaluating the performance of models in bearing fault detection, particularly in the early fault detection and identification of different fault types.

#### 3.1.3. Full-Cycle XJTU-SY Dearing Dataset

**Figure 4:**
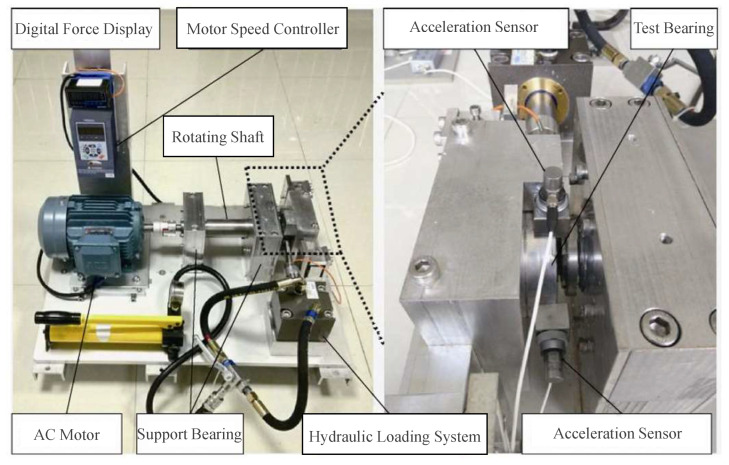
XJTU-SY bench.

The full-cycle XJTU-SY bearing fault data [31] are collected using the testing platform shown in Figure 4, which includes an AC motor, a motor speed controller, a support shaft, two support bearings, two accelerometers, and a test bearing. This testing platform is capable of conducting accelerated degradation testing, providing experimental data that closely reflect real-world conditions, and comprehensively capturing the entire lifecycle of bearings during the degradation process. The dataset used in this study includes vibration signals from three types of naturally occurring bearing faults: inner race faults (IF), outer race faults (OF), and cage faults (CF). Detailed data are presented in Table 6. The vibration signals are collected via accelerometers at a sampling frequency of 25.6 kHz, with a sampling interval of 1 min and a duration of 1.28 s for each sampling event.

## 4. Data Preprocessing

For the CWRU dataset, normal samples under all operating conditions are divided into training, testing, and validation sets at a ratio of 7:2:1. The training set is used for model training, whereas the validation set is used to evaluate the model performance during training. Subsequently, the detection effectiveness of the model is tested using a testing set that included normal and various types of abnormal data under different operating conditions. For the full-cycle IMS and XJTU-SY, only normal sample files are used as the training set, whereas the remaining files are used for testing. Each sample uniformly contains 2048 data points.

## 5. Experimental Results and Discussion

### 5.1. Results on Abnormal Bearing Dataset

In this section, we select samples from the CWRU dataset to evaluate the capability of the proposed model for bearing anomaly detection under multiple operating conditions and compare it with several classic supervised networks. The experimental results for the multi-condition CWRU dataset are presented in Table 7.

Our proposed self-supervised model demonstrates a high accuracy rate in detecting ten abnormal states under multiple conditions, with an average accuracy surpassing that of supervised neural networks (NNs) and deep neural networks (DNNs), which utilize negative samples. Although its performance is only slightly lower than that of supervised CNN and deep convolutional neural networks (DCNNs), as a self-supervised model that does not rely on labels, achieving performance close to that of some supervised models represents a significant advancement. This indicates that the proposed model can effectively reflect system changes under multiple conditions. A learning strategy that combines global and local scales allows the model to capture data features more comprehensively for the detection of anomalies in bearings under various conditions, thus improving the precision of early anomaly detection in the full-cycle dataset.

**Figure 5:**
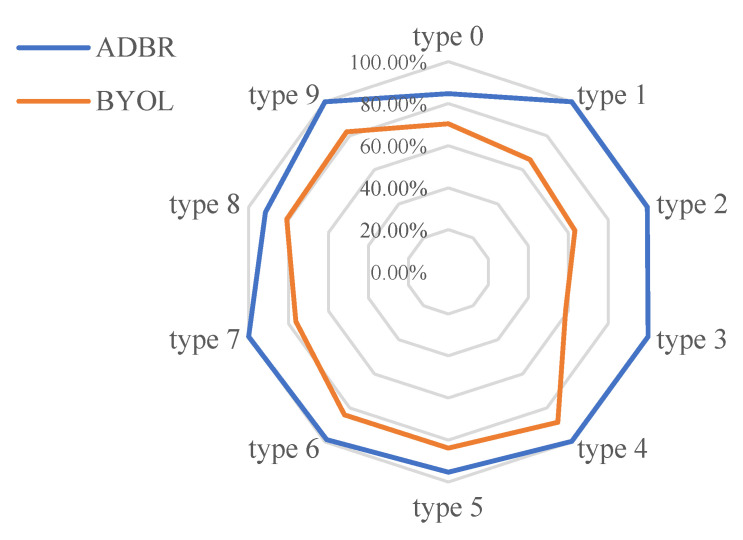
Accuracy of the two models for different anomaly detections.

To illustrate the effectiveness of the combined global- and local-scale learning strategy visually, we conduct an ablation experiment comparing the anomaly detection capabilities of our proposed ADBR model with those of the BYOL model alone under multiple conditions. We train both models on identical training sets and employ a uniform data preprocessing method to evaluate their performance on the same test set, allowing us to compare the contribution of the reconstruction loss. Figure 5 shows the accuracy rates of the two models for bearings with different types of losses under multiple conditions.

The results show that our method significantly outperforms the detection accuracy of various anomaly states under multiple operating conditions compared to the performance achieved by using only the BYOL network architecture. Experiments demonstrated that eliminating the reconstruction structure leads to a substantial decrease in accuracy, which more intuitively illustrates how utilizing the reconstruction loss enhances the model’s focus on local scales, thereby contributing to improved anomaly detection accuracy across multiple conditions.

To evaluate the impact of the weight coefficient α for contrastive learning loss and reconstruction loss on model performance, we design a series of experiments to analyze the effects of different α values on the model’s performance.

During the training process, we select different α values ranging from [0.1:0.1:0.9] to balance the weights of the contrastive learning loss and reconstruction loss.

**Figure 6:**
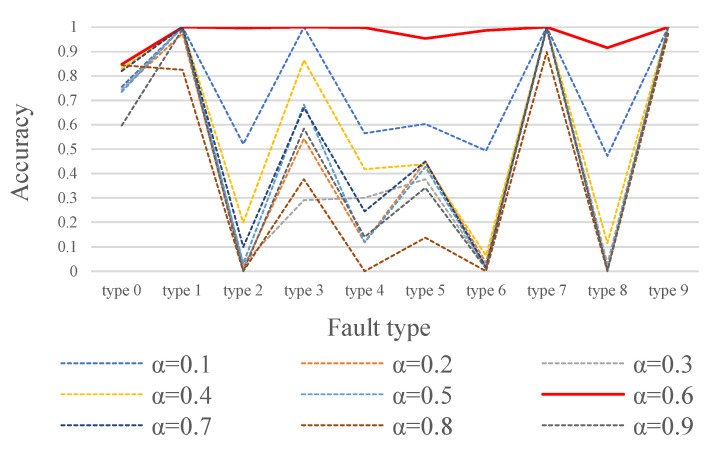
Impact of α on model effectiveness.

As shown in Figure 6, when the loss weight coefficient α of the ADBR model is set to 0.6, the model achieves the highest accuracy in detecting various bearing anomalies, demonstrating optimal model performance. Based on the experimental results, we select α=0.6 as the loss weight coefficient for the ADBR model, with the corresponding loss function formulated as follows:

$$L=0.6L_{\mathrm{contra}}+0.4L_{\mathrm{recon}}.$$

### 5.2. Results on Full-Cycle Bearing Dataset

In this section, we validate and compare the early anomaly detection capability of our model for bearings through experimental results on a full-cycle dataset, while also assessing the generalization ability of the model.

#### 5.2.1. Results on the IMS Dataset

**Figure 7:**
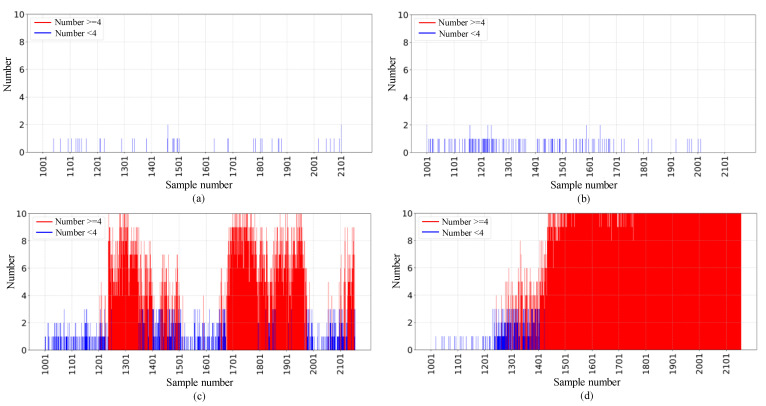
Result on dataset 1 of (**a**) bearing1_1, (**b**) bearing1_2, (**c**) bearing1_3, (**d**) bearing1_4.

**Figure 8:**
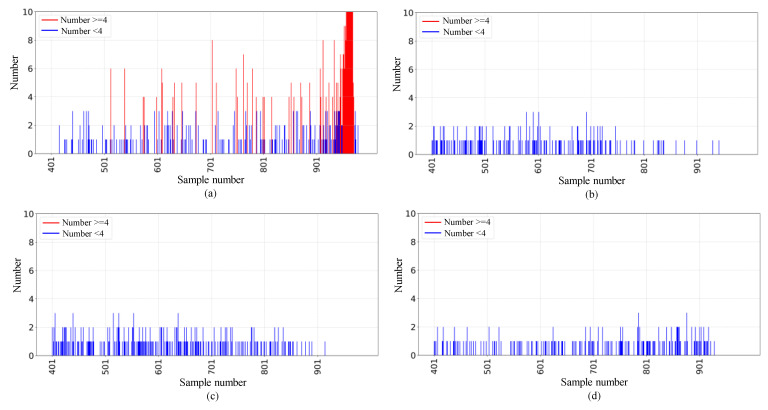
Result on dataset 2 of (**a**) bearing2_1, (**b**) bearing2_2, (**c**) bearing2_3, (**d**) bearing2_4.

Each sample contains 2048 data points; therefore, a single time file may contain multiple samples. We conduct experiments on two sets of full-cycle data from the IMS dataset and compare the results for normal and abnormal bearings. The results indicate that when the number of abnormal samples in a data folder is greater than or equal to four, the bearing is considered abnormal. Figure 7 shows that in the first set of IMS data, the number of abnormal samples in all time files of the first and second bearings is below the threshold, and no anomalies are detected. An anomaly is detected in the 1206th time file of the third bearing and the 1416th time file of the fourth bearing, while the other two normal bearings show no anomalies. These experimental results are fully consistent with the final state of the bearings. Figure 8 shows that in the second set of IMS data, an anomaly is detected in the 513th time file of the first bearing, while the number of abnormal samples in all time files of the other three bearings is below the threshold, and no anomalies are detected, classifying them as normal. This outcome also aligns with the final state of the bearings in the dataset.

**Figure 9:**
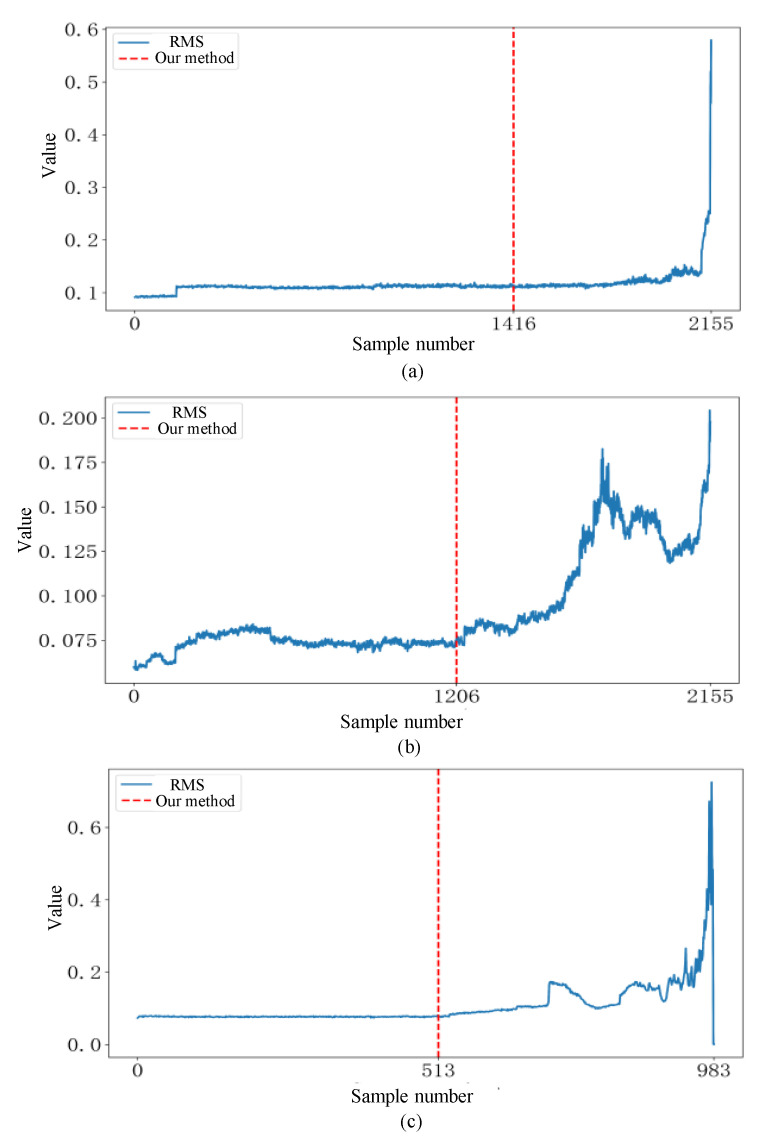
Comparison of the results of our method with the RMS curves for (**a**) bearing1_3, (**b**) bearing1_4, and (**c**) bearing2_1.

The detected final states of the bearings are aligned with the final states provided by the dataset. However, to assess the reliability of the early anomaly points identified by the ADBR model, the root-mean-square (RMS) method is used for verification. RMS is sensitive to changes in energy within the vibration signals because bearing abnormalities are typically associated with increases in the amplitude of the vibration signals. For instance, wear, pitting, or cracks in the rolling elements or inner and outer rings can lead to a significant increase in the energy of the vibration signal, resulting in a corresponding increase in RMS values. Therefore, RMS is effective in detecting changes in bearing conditions. We compare the anomaly detection results of these three bearings with their RMS curves as shown in Figure 9. We observe that our model successfully detects bearing anomalies in all cases before significant changes occur in the RMS curves. This demonstrates that our method is capable of detecting anomalies prior to the significant deterioration of bearing conditions (manifested as a notable increase in RMS values), indicating that its detection results are highly reliable. Such reliability is crucial for decision-making support in practical industrial applications.

To further validate the performance of our proposed ADBR model compared to other anomaly-detection methods, we conduct comparative experiments on the IMS dataset, specifically the Bearing2_1 dataset, using the early anomaly-detection methods listed in Table 8.

**Figure 10:**
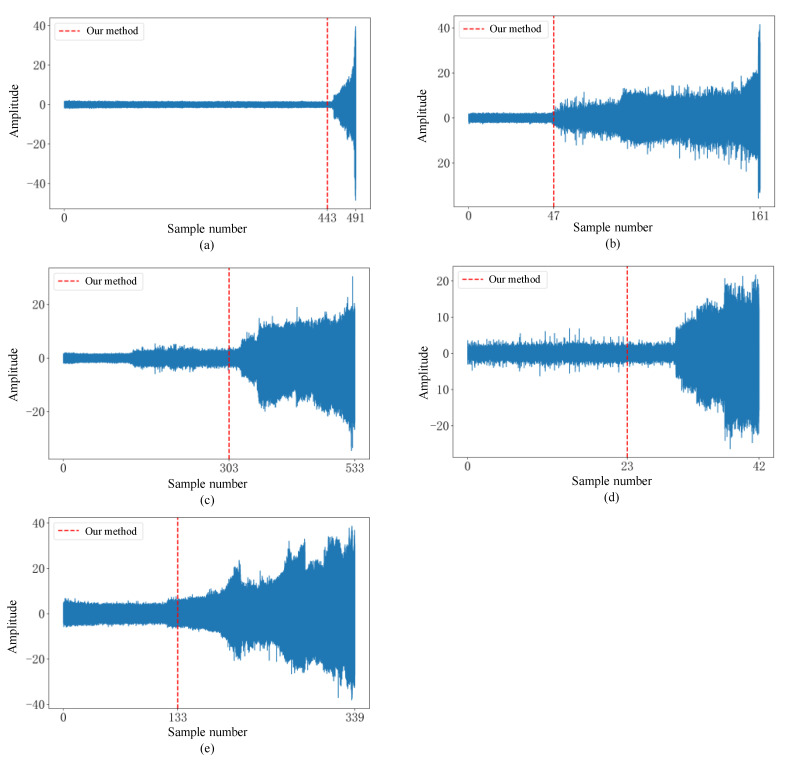
Comparison of the results of our method with the amplitude diagrams of (**a**) bearing2_1, (**b**) bearing2_2, (**c**) bearing2_3, (**d**) bearing2_4, and (**e**) bearing2_5.

Among these, methods 1–3 [33,34,35] are classical anomaly detection techniques, while methods 4–12 employ a combination of feature extraction and detection. Methods 13–17 are unsupervised anomaly detection methods similar to our approach, and Figure 10 illustrates the locations of the anomaly samples detected by various anomaly detection methods on Bearing2_1 of the IMS dataset.

Based on the results in Table 8, we find that the ADBR model detects an anomaly in the 513th data file, which occurs after 85.5 h of testing. This result is at least 50 data files (approximately 8.3 h) earlier than those of classical anomaly detection methods. In comparison, methods that combine feature extraction and detection identify early bearing anomalies in at least 22 data files (approximately 3.6 h). Additionally, compared to similar popular unsupervised models, the ADBR model detects early bearing anomalies in at least 14 data files (approximately 2.3 h). These results are obtained under consistent experimental conditions by using a publicly available dataset for early bearing anomaly detection. Therefore, it can be concluded that by integrating global- and local-scale learning strategies, the model can capture data features more comprehensively in anomaly detection, leading to the superior performance of our ADBR model compared to other unsupervised models in detecting early-bearing anomalies.

#### 5.2.2. Results on the XJTU-SY Dataset

This section further validates the generalization capability of the proposed model using another full-cycle bearing dataset, XJTU-SY, to comprehensively evaluate the performance of the model under prolonged operating conditions. For abnormal bearings, the amplitude gradually increases over time, and this significant increase in amplitude is a typical signal of equipment anomalies, indicating a sharp increase in vibration intensity at the onset of failure. Therefore, we compare the model results with the amplitude plots. As shown in Figure 10, the ADBR model detects anomalies before these changes became apparent. Therefore, in two early anomaly detection experiments conducted on two full-cycle datasets, the model successfully detects early anomalies in the bearings, which not only demonstrates its superior early anomaly detection ability compared to similar models but also verifies its generalization capability.

## 6. Conclusions

Based on the findings of this study, we propose a novel early anomaly detection model for bearings, named ADBR. The model combines a BYOL-based network structure with a reconstruction approach, enabling it to learn data features across multiple operating conditions at both the global and local scales, while being trained solely on positive samples. This approach effectively addresses two key challenges in industrial anomaly detection: the lack of negative samples and the frequent changes in the operating environment of the equipment. Experimental results on the multi-condition CWRU anomaly bearing dataset show that the ADBR model achieves an average accuracy of 96.97% in detecting various bearing anomaly states, demonstrating its great potential for early anomaly detection. Notably, when applied to the IMS dataset, the ADBR model detects faults at least 2.3 h earlier than traditional algorithms and other unsupervised methods, highlighting its significant advantage in early fault prediction. Finally, the model’s generalization ability is further validated using the XJTU-SY dataset. Future work will focus on exploring the impact of additional operational factors, such as lubrication and temperature variations, on the bearing fault detection model. These factors can significantly influence the operating conditions and fault characteristics of the equipment. By incorporating them, the model’s robustness can be further enhanced, enabling it to maintain high efficiency in fault detection even under complex and dynamic industrial environments. Moreover, accounting for these environmental factors will broaden the model’s applicability, allowing it to better handle bearing fault diagnosis tasks across a wider range of operational conditions, thus increasing its value and reliability in practical industrial applications.

## Figures and Tables

**Table 1:** List of symbols and their definitions.

Symbol	Definition
$D_{\mathrm{normal}}$	The raw time-series data from normal samples
$X_{i}$	A sample from the time-series data, where $X_{i}\in R^{t}$
ψ(t)	The Ricker wavelet function, defined in Equation (1)
$P_{i}$	The time–frequency representation of the sample $X_{i}$ after the Ricker
	wavelet transform
$g_{\theta }$	The projection head of the online network
$q_{\theta }$	The prediction head of the online network
$L_{\mathrm{contra}}$	The contrastive loss, defined in Equation (4)
$L_{\mathrm{recon}}$	The reconstruction loss, defined in Equation (5)
L	The overall objective function, defined in Equation (6)

**Table 2:** Training environment configuration.

Component	Configuration
Operating System	Windows 10
CPU	12th Gen Intel(R) Core(TM) i7-12700K
GPU	NVIDIA GeForce RTX 3090
RAM	32 GB
Storage	HS-SSD-A4000 2048G
Python Version	Python 3.8.18
Deep Learning Framework	PyTorch 2.3.0
CUDA Version	11.8

**Table 3:** Training parameters of the ADBR model.

Parameter	Value
Encoder	ResNet-18
Loss	MSE
Optimizer	Adam
Learning Rate	0.0003
Epoch	200
Batch Size	64
Shuffle	True

**Table 4:** Ten types of anomaly.

Type	Condition Type	Defect Severity
0	Normal	0
1	IRF	0.007
2	IRF	0.014
3	IRF	0.021
4	BF	0.007
5	BF	0.014
6	BF	0.021
7	ORF	0.007
8	ORF	0.014
9	ORF	0.021

**Table 5:** Details of the IMS dataset.

Date Set	Number of Files	Number of Positive Sample Files	Abnormal Bearings
Dataset 1	2156	1000	1_3 and 1_4
Dataset 2	984	400	2_1

**Table 6:** Details of the XJTU-SY dataset.

Bearing Dataset	Number of Files	Number of Positive Sample Files	Fault Element
Bearing 2_1	491	315	Inner race
Bearing 2_2	161	30	Outer race
Bearing 2_3	533	225	Cage
Bearing 2_4	42	20	Outer race
Bearing 2_5	339	95	Outer race

**Table 7:** Comprehensive comparison of classification accuracy of different fault diagnosis methods.

Method	Fault Type										Average (%)
	0 (%)	1 (%)	2 (%)	3 (%)	4 (%)	5 (%)	6 (%)	7 (%)	8 (%)	9 (%)	
NN	100	80.75	65.50	100	82.25	87.25	79.75	76.50	99.50	76	84.75
DNN	100	97.50	92	100	94.50	94.50	95.50	92.75	99.50	83.25	94.95
CNN	100	97.75	90.50	100	99.50	94.25	98.25	99.75	100	96.25	97.63
DCNN [32]	100	100	99.50	100	100	100	100	100	99.75	100	99.93
ADBR	84.76	100	99.58	100	99.79	95.34	98.73	100	91.53	100	96.97

**Table 8:** Results on Bearing2_1 by various anomaly detection methods.

Method	Results	Method	Results	Method	Results
1.KNN	563	7.RMS + LOF	535	13.DIDAD [19]	533
2.LOF	609	8.Kurtosis + LOF	700	14.ANOGAN	533
3.one-class SVM	609	9.SDAE + LOF	700	15.USSCNN [20]	531
4.RMS + SVDD	535	10.RMS + iForest	535	16.UODA	531
5.Kurtosis + SVDD	703	11.Kurtosis + iForest	650	17.FDDA [22]	527
6.SDAE + SVDD	600	12.SDAE + iForest	720	18.ADBR	513

## Data Availability

The CRWU dataset, the IMS dataset, and the XJTU-SY dataset are used in this article (all three datasets are open source on the web).
